# The Foreign Journals: Pathology, Practical Medicine, and Therapeutics

**Published:** 1842-01

**Authors:** 


### PATHOLOGY, PRACTICAL MEDICINE, AND THERAPEUTICS.

*A Case in which an Abscess behind the Œsophagus was mistaken for Œdema of the Glottis, and occasioned death by Asphyxia.* By Dr. BALLOT, Physician to the Hospital of Gien.

THIS is not a common occurrence, though cases of it are on record both in French and English writings. The patient whose history M. Ballot relates was a man 40 years of age, of intemperate habits, and much exposed by his employment to all changes in the weather. He had suffered for some days from sore throat, when on Sept. 27, 1837, he applied at the hospital.

At that time the pharynx was slightly red and dry, but not perceptibly swollen; deglutition was difficult, and the patient was unable to speak aloud. He complained of pain in the pharynx, and of a sensation as of some foreign body there, impeding deglutition and respiration. The finger introduced deep into the pharynx distinguished a tense and elastic swelling, on a level with the upper part of the larynx, apparently continuous with the glottis, and perceptibly diminishing its aperture.

The patient was bled, to 18 oz. by weight, and was put on a strictly antiphlogistic plan of treatment. On the following day venesection was repeated to the same amount, thirty leeches were applied to the throat, and a blister was put on the back of the neck. These measures were not followed by any amendment, paroxysms of threatening suffocation occurred, but deglutition was not observed to be more difficult on the 29th than it had been at the time of the patient's admission into the hospital. The patient continued to be treated very actively until the 8th of October, when laryngotomy was performed in order to relieve him from the suffocation with which he appeared to be threatened every moment.

The opening into the larynx did not produce any marked improvement, but the patient breathed freely after the introduction of a canula into the trachea. During the night, however, the tube became displaced, and in the morning the man was found asphyxiated.

On examining the body after death it was found that no tumefaction of the glottis existed, but that its orifice was almost completely closed by a fluctuating swelling of the size of a hazle nut which projected over its upper part. It extended downwards below the cricoid cartilage, and compressed the cavity of the larynx considerably. It was formed by a collection of healthy pus, which was in contact with the anterior wall of the vertebral column and the posterior surface of the œsophagus, and extended along the sides of the larynx, in such a manner that on the left side it was not more than a quarter of an inch from the upper angle of the incision made between the thyroid and cricoid cartilages. These circumstances explain the prominence felt by the finger passed into the pharynx, as also the reasons why respiration was so much impeded, and why the canula introduced into the opening had such a constant tendency to become displaced. They further suggest that in any similar case tracheotomy should be preferred to laryngotomy.

*Archives Générales de Médecine. Octobre,* 1841.

---

*On a Peculiar Form of Diarrhœa which attack Stokers on board Steam-boats.* By Dr. J. J. Renault, Surgeon to the (French) Government Steamer Minos.

The exceedingly high temperature to which these men are exposed in the performance of their duties, with the cold to which they are often subjected whenever they come on deck concur to render their occupation exceedingly unwholesome, and usually shorten their lives very considerably.

They seem to be liable to a peculiar kind of diarrhœa, which attacks those who had apparently been in the enjoyment of good health. A vague sense of faintness, and occasional shivering, are followed by a sudden desire to relieve the bowels, and the patient after voiding a liquid stool feels much relieved, and is able to go about his occupation till the symptoms recur. The intervals between each attack become shorter, and the diarrhœa continues sometimes only for twenty-four hours, at other times for three, four, or five days, but never beyond this period. The frequency of the stools likewise varies from five to twenty in the twenty-four hours. At first they consist of true feces mixed with mucus, afterwards of a viscid mucus alone, or even are quite watery. This diarrhœa is not attended by pain or colic, the appetite is not even diminished at the commencemeut of the attack, but afterwards the thirst becomes very great, and more or less complete anorexia ensues.

The disease always terminates in convalescence, but in a few cases a mild form of enteritis supervened, requiring local depletion for its cure. A low diet and opium are the two means by which M. Renault combats the disease, which is important only from its very frequent occurrence, and debilitating effects.

[We suspect that M. Renault takes a generalizing flight somewhat too wide from his premises: but we insert this paper chiefly with the view of calling the attention of the surgeons of our steam-boats to the circumstance.]

*Revue Médicale. Août,* 1841.

---

*On the Action of certain varieties of Ranunculus, when used as external applications.* By Dr. G. Polli.

The ranunculus is frequently employed by the country-people and quack-doctors in different parts of Italy. Dr. Polli had frequently used it empirically in obstinate rheumatic affections and in some instances with such good success that he determined to examine which varieties of the plant were the most powerful, and in what manner the remedy could be best applied.

His experiments with the plants given internally are but very few, but he has ascertained that applied externally the *ranunculus sceleratus* is the most active, then the *acris*, the *bulbosus*, and the *flammula.* The leaf and stem of the *sceleratus* and *acris* are the chief seat of the active principle of those plants, but the powers of the *bulbosus* reside chiefly in the root and stem, and those of the *flammula* in the flower. The summer and autumn are the seasons when the plants possess their virtues in the highest degree, but from the end of December to the end of March they are almost completely powerless.

The fresh plant bruised and applied to the skin is a powerful irritant, producing either a general redness of the skin, or an eruption of miliary vesicles, or blistering the part, and producing a sore which does not heal for fourteen or fifteen days. An essential oil, a vinegar, tincture, spirit, and distilled water which may be prepared from the recent plant, preserve its peculiar properties.

The diseases in which the remedy is now used by Dr. Polli are chronic sciatica, various forms of dyspepsia and gastralgia, and cases in which chronic irritation exists about the larynx or trachea. In cases of sciatica he covers the heel either with the recent plant bruised, or with compresses dipped in some liquid preparation of it, which he allows to remain in contact with the part for twelve hours. At the end of this time he removes the application: in the course of the ensuing twelve hours, the patient experiences considerable pain in the heel which is mitigated by the application of warm poultices, and ceases on the formation of a blister which usually contains a large quantity of serum. In some instances a second application of the remedy to the lower part of the loins is necessary, though very often the first application to the heel is sufficient to effect a cure. Of thirty cases of chronic sciatica thus treated, Dr. Polli assures us that not one resisted the second employment of this remedy.

*Annali Universali di Medicina. Decembre,* 1840.

---

*On a Peculiar Morbid Parasitic Formation with specific organized Seminal Corpuscles.* By Professor Müller.

The disease here described was first observed in the cellular tissue of the orbit of a young pike, in the substance of the sclerotica, and between it and the choroid. It consists of cysts from one fifth to one half of a line in diameter, with very delicate walls, and containing a whitish substance which is composed of very small granules with molecular motion, and of innumerable corpuscles very like spermatozoa, but quite motionless. These corpuscles have an oval body and a tail. The body has a general resemblance to an elliptical blood-globule, and is about as large as the blood-globule of the pike, like which also it has two surfaces and a thick edge. In its interior there are always at the end opposite to that whence the tail proceeds, two rather long vesicles whose smaller ends converging at the end of the corpuscle are there united, and thence lying symmetrically in it, gradually diverge to their larger extremities. Besides these vesicles the corpuscles contain a transparent fluid. The tail of the corpuscle is a filament like those of spermatozoa, three or four times as long as the oval part and sometimes bifurcated. It appears to be a prolongation of the wall of the corpuscle ; the cavity of the latter is not continued into it.

In the pike, the cysts with such corpuscles as these, are found only in the orbit; but in several other river-fish cutaneous eruptions are sometimes met with, consisting of vesicles with corpuscles differing from those just described, in not being caudate, but resembling them in containing the two oval diverging vesicles; and since his first observations were made, the author has found them in cutaneous eruptions in a number of fish, as well in those of his own country as in those of other parts of Europe, Brazil, North America, the Nile, &c. On the skin the vesicles are most common in the *zander*, (of which the author does not give the Latin name, but which we find translated as the perch-pike;) and in this the mode of development of the corpuscles has been observed, for among a great number of them there are found some which show the two diverging vesicles separated and enlarged, and lying free in the interior of the corpuscle, and other examples in which two corpuscles lie near one another, surrounded by one very pale cell, and presenting each all the characters of the single corpuscles, together with the rudiments of diverging vesicles in each. So that the mode of development of the corpuscles is in all probability this: the diverging vesicles are the germs of new corpuscles; and as they come to be developed they swell up, separate, and lie in pairs within the cavity of the corpuscle, which is transformed into the thin-walled cell containing them. Then as they are yet further developed, the maternal cell dissolves and they become free, each as a separate corpuscle.

The author regards this as a remarkable example of the propagation of a disease by a living *seminium morbi*: it seems to hold an intermediate place between the acknowledged parasitic entozoa and epizoa, increasing by distinct ova, and the cells of ordinary morbid structures increasing by the development of their nuclei, and of one cell within the other.

*Müller's Archiv.* Heft v. 1841.

### *On the New Etiology of Tinea.* By M. GIBERT.

IN his last lecture at the Hospital St. Louis, in treating on tinea, M. Gibert referred to the opinion of M. Gruby, of Vienna, that the true tinea—*favus* of Alibert, *porrigo lupinosa* of Bateman—was owing to the development of a sort of mould or parasitic vegetable production, the granules of which are capable of reproduction, and may transmit the disease from one individual to another. Two young sisters presented at the lecture of M. Gibert had some crusts of true tinea on the shoulders and subclavicular region, which, when examined by the microscope, offered the appearance of regular vegetable organization, consisting of small yellowish lobules, some of which were still attached to a fragment of stalk. These young girls had evidently contracted the disease by contagion. M. Gibert is of opinion that we can explain many of the peculiarities of the history of tinea by admitting that it is caused and kept up by a parasitic production, at the same time that the contagion (denied by some moderns, especially by Alibert) is placed beyond doubt. Thus the regular form of the crusts of favus; the transmission by granules which are detached and constitute true vegetable germs; the production of tinea in children and weak subjects in whom the skin is accessible to parasites; the continuity of the disease which is perpetuated by a species of vegetation peculiar to itself; the superposition of the crusts which may be detached from the skin without encroaching upon it, and which are reproduced after a certain time; the necessity of attacking the disease by local means capable of destroying the life of the vegetable parasite, are circumstances all of which may be easily explained by the hypothesis of Dr. Gruby.

It is necessary to distinguish the true *tinea favosa* from the *pseudo-tinea*, as these have been shown by microscopic observers to be entirely distinct species. (See Gibert's Traité Pratique des Maladies de la Peau, pp. 235 et seq.)

*Gazette Médicale de Paris. Août* 14, 1841.

M. Gruby has since asserted that he has discovered another plant in the bullæ of rupia.

*Gazette Médicale de Paris. Août* 24, 1841.

---

*On Rheumatic Dermalgia, or Rheumatism of the Skin.* By J. H. S. Beau, Physician to the Central Bureau of the Hospitals of Paris.

Neuralgia of the skin has hitherto been usually confounded with pains of the nervous trunks, muscles, &c. M. Piorry was the first who referred it to a separate head under the name of *dermalgia.* It frequently coexists with neuralgia of the nervous trunks, with ramollissement of the brain; or occurs in cases of inflammation of the spinal cord. Severe pain in the uterus is often attended with dermalgia of the skin of the pelvis and thighs, and clavus hystericus is frequently a neuralgic affection of the skin.

There are several other forms of this affection, but one which has escaped notice down to the present time is rheumatic dermalgia. This is of more frequent occurrence among men than women, and is induced by damp, cold, and those other causes to which rheumatism generally is owing. Hence it is most common at the beginning of spring. The head and lower extremities are the parts usually attacked, but the pain is not stationary in one place; often changing its seat in a gradual manner, just as erysipelas sometimes wanders from place to place. Patients experience two kinds of pain, the one abiding, the other intermittent and severe, resembling the prick of a pin or an electric shock, and recurring about every thirty seconds. The abiding pain is frequently little more than a permanent exaltation of the natural sensibility of the skin. Friction of the part with the finger or with the patient's dress, always increases the pain; and if the affected part is covered with hair, very severe suffering may be produced by passing the hand over the hair. The intermittent pain is often at once excited by touching the part in this manner, and though firmer pressure puts a stop to the permanent pain, the return of the intermittent pain cannot be thus prevented. The intermittent pain is always considerably worse at night. Rheumatism of the skin usually alternates with that form of the disease which affects the muscular and fibrous tissues. Its usual duration is from a day to a couple of days, and it subsides by degrees just in the same way as it made its attack. The author met with three instances in which it was accompanied with fever and involved a much larger surface of skin than usual. It is, in general, an affection easily curable. The indications for its treatment do not differ from those to be observed in ordinary rheumatism, but it does not generally require any very active remedial measures. To prevent its recurrence, it is always desirable for the patient to wear flannel next his skin.

*Archives Générales de Médecine. Septembre,* 1841.

---

*On the Mortality of Leeches after they have been used.* By M. Varlitz, one of the assistants in the clinical wards of the hospital at St. Petersburg.

Experiments were instituted during the years 1839-40, under the direction of Professor Zeidlitz, with the view of ascertaining the influence which the blood of man when labouring under different diseases exercises on the health of leeches.

The leeches after being applied to any patient were placed by themselves in a cylindrical glass vessel, with a large mouth, and capable of holding from half a pint to two pints of water. The date of their being placed there, and the disease of the person to whom they had been applied were carefully noted, they were supplied regularly with fresh water, and were not mixed with other leeches till after the lapse of three months.

The vessels in which they were kept appear to be much more favorable to their health than those made of earth, since only 23 per cent. died when placed in the former, while 90 per cent. died of those which were kept in earthen vessels. Of 455 leeches employed during ten months, from Sep. 1839, to July 1840, 225 or 48 per cent. died, and this mortality is probably almost entirely owing to the noxious properties of the blood they drew, since 50 per cent. died of those who did not disgorge the blood, while the mortality of those who were emptied of blood before being placed in the vessel was much less.

Those who were put by, without being emptied, disgorged the blood sooner or later; most speedily (within the second or third day) when they had been applied to hemorrhoids, or to patients suffering from scurvy, or typhus.

The proportionate mortality of leeches employed in different diseases was as follows:

| | | | | | |
|---|---|---|---|---|---|
| Of 60 | leeches employed in cases of | Typhus | there died | 42 = 70 | per cent. |
| 176 | ,, | Hemorrhoids | ,, | 72 = 40 | ,, |
| 93 | ,, | Nervous Diseases | ,, | 36 = 38 | ,, |
| 35 | ,, | Pulmonary Phthisis | ,, | 13 = 37 | ,, |

*Journal de Médecine, &c. de l'Académie Impériale Médico-Chirurgicale de S. Petersbourg.* Second Cahier, 1841.

---

### *On a Case of Paralysis of the Two Facial Nerves.* By M. CONSTANTIN JAMES.

THE chief interest of this case is its illustration of the antagonism between the muscles of the two sides of the face. The patient was a young and otherwise healthy lady, aged 22, in whom, without any evident cause, there occurred a complete and sudden paralysis of the left facial nerve with all the deformity of the features that usually accompanies it. She was treated by galvanism under the direction of M. Magendie, and at the end of the third week from the first seizure the deformity began to disappear and was shortly altogether lost. This however was not from a cure of the disease, but from paralysis having now seized the right facial nerve, and placed the muscles supplied by it in the same powerless condition as their fellows on the left side. The galvanism was continued on both sides of the face, and in about ten days the nerve of the left side began to recover its energy, and all the deformity returned, the features being drawn completely over to the left side. In ten days more the right nerve also began to recover, and as they now both increased in power, in the same proportion did the features regain their symmetry, till, when both the nerves had acquired their healthy state there was no deviation from the natural expression of the face.

*Gazette Médicale. Septembre* 18, 1841.

---

### *Transmission of Glanders from Man to Man.* By M. BERARD.

AT the sitting of the Institute of France, on November 15, M. Berard communicated the case of a medical student who had been for many days engaged in attending and dressing a patient in the Necker Hospital that had died of glanders, and who, at the p. m. examination, had his hands for a long time in contact with the ulcerated and gangrenous parts about the head and face. He had no wounds or scratches on his hands; but on the night after the examination, having for some little time had slight diarrhœa, he awoke with shivering, which was soon followed by slight fever and general uneasiness. For two days he continued unwell but able to get up, but on the third the pain became more severe, and settled in the left thigh, the right shoulder, and the right side of the chest. On the fifth day, tumours like 'farcy-balls' appeared in the thigh and shoulder, and some days after, one being opened, discharged pus mixed with blood, which, inoculated into a horse, produced glanders that proved fatal on the same day. Another tumour now appeared over the right ancle and suppurated. And lastly, fourteen days from the beginning of the disease, the skin of the nose became red, hot, and painful; on the fifteenth, the redness had extended to the cheeks, the eyelids, and the middle of the forehead, and gangrenous blisters and pustules appeared here and there upon the face. On the sixteenth day, these signs were yet more marked, and a bloody fluid flowed abundantly from the nose; pustules came out over every part of the body, and the patient died in the course of the night.

This case, M. Berard remarks, proves that glanders may be communicated by miasmatic infection; for the patient had no sore places on his fingers during all the time that he was attending the case from which he received the disease; he neither pricked nor cut himself at the autopsy, and he always took care to wash his hands after handling the infected matter.

[We do not think the miasmatic infection proved. It is certain that persons may be poisoned by animal matters absorbed through the sound cuticle when they have lain long in contact with it; many have been thus affected after dissections in which, with sound hands, they only handled the diseased parts. M. Berard is wrong in regarding this as the first case of glanders communicated from man to man. A nurse at St. Bartholomew's Hospital died of the disease received from a patient there; the case was published by Mr. Brush in the London Medical Gazette, April 24, 1840.]

*Gazette Médicale. Nov.* 20, 1841.

---

*On the Repeated Application of one or two Leeches to the Knee in Dysmenorrhœa.* By M. Trousseau.

In three hospital patients under the care of M. Trousseau the catamenia have followed the application of a leech to the internal surface of the knee. In one case a leech was applied to the right knee; while it held on the patient experienced nothing particular, but as soon as it fell off pains in the loins came on, which lasted almost an hour, and the discharge then appeared. The next day it was arrested again, and a leech was applied to the left knee; and the discharge appeared as before, and continued as usual during three days. In another case the pains of uterine congestion commenced with the application of the leech, which adhered during an hour. The effect produced by one leech is not wonderful, says M. Trousseau, because if the bleeding is allowed to continue, as large a quantity of blood flows as the ordinary amount of menstrual discharge.

*Bulletin Général de Thérapeutique. Février* 15 *et* 28, 1841.

---

*Case of Hepato-pleuro-bronchial Fistula.* By M. Pelletan, Jun.

At the sitting of the Royal Academy of Medicine, March 23, 1841, M. Pelletan presented the lung, pleura, and liver of a man who died under his care in the hospital of St. Louis. Some time before the death of this patient, he began to expectorate a yellow matter, which had some resemblance to bile, but accident prevented it from being analysed. On post-mortem examination it was found that an abscess had broken through the whole thickness of the right lobe of the liver and the diaphragm, and communicated with the pleura, which formed a pouch at this situation. The neighbouring portion of lung was condensed, and a fistulous canal passed through the pulmonary substance and communicated with the bronchial tubes.

*Gazette Médicale de Paris. Mars* 27, 1841.

